# Medical and Philosophical News

**Published:** 1781-04

**Authors:** 


The late Dr. Fothergill, a little before his death, was preſent on a ſurvey at Buxton with two other phyſicians. As the Duke of Devonſhire means to make ſome improvements at that place, they were deſirous of propoſing to his Grace, ſuch as might prove conducive to health. —At preſent there are only two baths at Buxton, one for gentlemen, the other for ladies; and four perſons only can bathe in each at a time. The rule for bathing is to ſet apart the firſt hour for the gentlemen and ladies who lodge lodge in the houſe, the ſecond for thoſe who lodge in the town, and the third for the inhabitants. The preference thus given to thoſe who lodge in the houſe, very often occaſions ſo many to be crowded together in one apartment, that they ſuffer as much from this circumſtance, as they gain benefit from bathing. The ſcheme in agitation, therefore, is for every houſe to be furniſhed with a bath, that it may be no longer a monopoly.— As the natural heat of the Buxton water is only about 80° of Fahrenheit's thermometer, Dr. Fothergill propoſed, as a farther improvement, that it ſhould be heated artificially to about 90°, in order to render it more efficacious in cutaneous diſeaſes.

